# Risk Factors for Dental Erosion After Bariatric Surgery: A Patient Survey

**DOI:** 10.1016/j.identj.2021.11.001

**Published:** 2021-12-20

**Authors:** Fatimah Alsuhaibani, Abdulaziz Alsuhaibani, Dan Ericson, Kerstin Larsson

**Affiliations:** a Department of Restorative Dentistry, College of Dentistry, Qassim University, Buraydah, Saudi Arabia; b Department of General Surgery, King Saud Medical City, Riyadh, Saudi Arabia; c Department of Cariology, Faculty of Odontology, Malmö University, Malmö, Sweden

**Keywords:** Bariatric surgery, Dental erosion, Oral health

## Abstract

**Introduction:**

Treatment of obesity by bariatric surgery has increased in recent years. Reported side effects that may predispose to dental erosion include reflux, vomiting, and an increased frequency of intake of food and drink.

**Objective:**

The aim was to investigate long-term dietary behaviour and experiences related to symptoms of dental erosion at least 5 years after bariatric surgery.

**Methods:**

An online questionnaire study was conducted amongst 250 patients who had undergone bariatric surgery at King Saud Medical City in Saudi Arabia 5 years ago or more. It comprised 36 questions on demographic data, dietary habits, general health, dental health, and oral symptoms. The data were analysed using Chi-square and sign tests (significance level *P* < .05).

**Results:**

A significant increase in acidic reflux and vomiting was found after bariatric surgery and appeared to increase with time after surgery. Also, a significant association between presence of acidic reflux and symptoms of dental erosion was found. However, 68.5% reported improved overall well-being after surgery. The response rate was 21.6% (most were female, aged 30-59 years). Respondents were generally not advised to visit a dentist in connection with bariatric surgery.

**Conclusions:**

This long-term cross-sectional study suggests a time-dependent, increasing occurrence of vomiting and acidic reflux after bariatric surgery. Vomiting and reflux became even more common after 5 to 10 years. A significant relationship emerged between a high frequency of acidic reflux and a high frequency of oral symptoms related to dental erosion. Daily occurrence of general symptoms related to dumping syndrome were reported by the majority. However, in a 5- to 10-year perspective, general symptoms related to dumping syndrome and symptoms from dental erosion did not seem to detract from the respondents’ overall satisfaction with daily living. Oral health problems might be reduced if patients who had bariatric surgery were referred to a dentist for prevention and monitoring.

## Introduction

Bariatric surgery is a common procedure worldwide for the treatment of obesity and obesity-related diseases, such as diabetes mellitus, with increasing numbers of operations in recent years.^1^, ^2^, ^3^, ^4^ Also, obese patients are at risk for oral diseases such as dental caries,^5^ periodontal diseases, and tooth loss.^6^^,^^7^ The mechanisms underlying the weight loss achieved by different surgical techniques may vary; malabsorption could be achieved and be used by itself or in addition to restrictive procedures.^8^ A feeling of fullness at an earlier stage is often experienced.^9^^,^^10^ The side effects of those different methods could also be different.

Bariatric surgery is considered to be effective and safe^1^^,^^2^ and is reported to result in better management of chronic diseases as well as in substantial, lasting weight loss. Further, obese patients who have undergone bariatric surgery are reported to experience increased self-confidence, both socially and psychologically.^3^^,^^11^

However, bariatric surgery may also result in undesired general and oral effects. Complications include gastroesophageal reflux, malnutrition, anemia, dehydration, and vitamin and mineral deficiencies.^12^ Many patients experience multiple symptoms or discomfort, such as nausea, vomiting, feeling too full, abdominal cramps, diarrhoea, dizziness, or rapid heart rate after eating. This is referred to as dumping syndrome.^13^ Thus, the patients cannot eat as large meals as previously. Standard dietary recommendations for bariatric patients include small, frequent meals (4-6/d), thorough and slow chewing, and sipping fluids throughout the day.^14^ These frequent and prolonged mealtimes could be associated with increased risks to dental health, such as caries and dental erosion.

Vomiting and gastroesophageal reflux, which expose the oral cavity to gastric acids, are risk factors for dental wear, in particular dental erosion.^15^ In a short follow-up study (6 months), it was found that the severity of dental wear increased with time after bariatric surgery.^16^ This may be related to reflux as well as to chronic vomiting and changes in dietary pattern.^17^

Longer term, there is evidence of oral complications after bariatric surgery.^17^ One such complication, dental erosion, may not be attributable solely to an increase in prevalence of reflux. Preferences in what and how patients eat and drink/ingest may also have changed in the years following bariatric surgery. For instance, a higher intake of sweet foods has been reported.^18^ Eating habits of an unhealthy nature, such as this, have been discussed, and it has been suggested that eating behaviour does not improve after bariatric surgery, as there may still remain a need to experience the satisfaction associated with eating.^19^ It is also reported that after bariatric surgery, patients find it difficult to comply with dietary recommendations in the long term.^20^ It is therefore of interest to explore how patients report a drift in dietary habits more than 5 years after surgery. Do patients return to their old habits, are new habits introduced, and is there an increased risk of erosion?

Bariatric surgery has a significant impact on daily life and also influences oral health–related quality of life.^11^^,^^16^^,^^21^^,^^22^ Some disadvantages are reported, although patients report an overall improvement in quality of life. Self‐reported oral health problems such as caries and tooth wear were more common after bariatric surgery, which is in accordance with clinical studies.^23^, ^24^, ^25^ Oral health problems are more frequently perceived amongst individuals who have undergone bariatric surgery than amongst healthy obese and nonobese individuals.^22^ Bariatric surgery may thus be considered a risk marker for impaired oral health.

There are no reports assessing the long-term effects (5 years or more) of bariatric surgery on dietary and behavioural changes as risks for dental erosion. Considering the increasing prevalence of bariatric surgery,^4^ it is important to document patients’ experiences of dietary and behavioural changes which might in the long term predispose to development of dental erosion.

The aim of this study is to investigate long-term dietary behaviour and experiences related to symptoms of dental erosion in patients who had undergone bariatric surgery at King Saud Medical City (KSMC) in Riyadh, Saudi Arabia, at least 5 years previously.

## Materials and methods

This cross-sectional questionnaire study was designed after a literature review of related sources.^18^^,^^26^ The study protocol was approved by the institutional review board at KSMC in Riyadh, Saudi Arabia. Participation was voluntary.

### Subjects

All patients (N = 403) who had undergone bariatric surgery at KSMC 5 to 12 years ago (from 2008 to 2015) were selected. From this primary selection of 403 patients, valid contact information was available for 250 patients.

### Questionnaire

An online questionnaire (using Google Forms) was conducted, including direct questions, with one or more alternative answers to be chosen. The questionnaire (Appendix B) consisted of 5 sections related to demographic data, dietary habits, general health, dental health, and oral symptoms, all of which were considered relevant to bariatric surgery and dental erosion.

The demographic data included patients’ age, gender, and educational level. The dietary-related section compared patient-reported habits before and after the surgery, including questions about dietary intake, such as the number of meals per day, and consumption of sugar, acidic food, and beverages. General health questions comparing patient-perceived conditions before and after the surgery, such as experience of acidic reflux, vomiting, dry mouth, and symptoms related to dumping syndrome were included, as well as the patients’ weight before and after surgery. Respondents were also asked to describe their overall sense of well-being after bariatric surgery. Dental health–related questions included frequency of dental appointments and oral hygiene routines. The final section of the questionnaire concerned with postoperative oral symptoms, for example, hypersensitivity of the teeth.

Symptoms or discomfort such as nausea, vomiting, feeling too full, abdominal cramps, diarrhoea, dizziness, or rapid heart rate after eating were categorised, in tables and text, as dumping syndrome.

The questionnaire was translated into Arabic and tested in a pilot study with 3 patients who had undergone bariatric surgery and was adjusted according to the result, before being sent to patients. The pilot study patients’ data were not included in the study.

### Data collection

The eligible patients, for whom the only information relayed was their phone numbers, were invited to participate in the web-based questionnaire. First, they completed a consent form (Appendix A). The consent form, which was linked to the questionnaire, was sent electronically by the KSMC call centre, as a text message to the eligible patients’ mobile phones. If patients were unwilling to participate, a message of thanks appeared and the form was closed. Those patients who gave consent on the form and decided to participate continued to the second step by answering the questionnaire that appeared. The patients could terminate the questionnaire whenever they liked. Incomplete forms were excluded automatically.

In the questionnaire, no personal details or contact information were collected. The data did not include any information that could identify patients or link any personal information to any patient. Thus, the anonymity of the patients was maintained throughout the study.

### Data analysis

The reported frequency of acidic reflux and vomiting were categorised as “yes” by adding up the alternatives daily, weekly, and monthly, or “no” for the alternative never, for statistical analysis.

The frequency of acidic reflux and vomiting before and after bariatric surgery was compared for each individual and stratified as less or unchanged frequency or alternatively higher frequency.

The answers to questions Q5, Q7, Q9, Q11, and Q31-34 were considered background information on the population and were not further analysed. Q18 and Q21-23 were omitted from analysis as they were found to be unclear or unintentionally contradictory. Q36 was also omitted, as it was an open question answered by only half of the respondents. The collected responses did not add anything relevant for this study to what was previously conveyed in the fixed alternative questions (Appendix B).

### Statistical analysis

Chi-square test and sign test were used to determine any statistical differences between groups. A significant difference was set to *P* < .05.

## Results

The questionnaire was sent out to 250 subjects who had undergone bariatric surgery 5 or more years ago. There were 54 responses (17 men, 37 women), which corresponds to a response rate of 21.6%. The answers are tabulated in Tables 1 and 2.

**Table 1:** Demographic and surgery-related data, referring to questions numbers 1-4 and calculations from 27-29.

Demographic and surgery-related data	Variable	N = 54	%
Age	Younger than 20 years	-	-
	20-29 years	-	-
	30-39 years	23	43
	40-49 years	19	35
	50-59 years	11	20
	60 years or older	1	2
Gender	Male	17	31
	Female	37	69
Education	Primary school	2	4
	Middle school	-	-
	High school	9	17
	Diploma degree	10	18
	University degree	28	52
	MSC or higher degree	5	9
Postoperative time	5-7 years	21	39
	8-10 years	12	22
	>10 years	21	39
BMI before bariatric surgery	≥30	54	100
BMI after bariatric surgery	<30	27	50
	≥30	27	50

BMI, body mass index; MSC, Master of Science.

**Table 2:** Answering frequency to questionnaire.

Questions	Answers	No.	%
*Q5,* Eating or drinking per day	1-2 times	12	22
	3-4 times	28	52
	5-6 times	12	22
	>6 times	2	4
*Q6,* Eating or drinking nowadays compared to before bariatric surgery	Less frequent	31	57
	Same frequency	6	11
	More frequent	17	32
*Q7,* Drinking acidic drinks for example water or tea with lemon/soft drinks/energy drinks/juices per day	None at all	16	30
	1-3 times	20	37
	4-6 times	13	24
	>6 times	5	9
*Q8,* Frequency of drinking acidic drinks nowadays compared to before bariatric surgery	Less frequent	37	69
	Same frequency	10	18
	More frequent	7	13
*Q9,* Taking sugar in coffee/tea or eat sweets per day	None	5	9
	1-3 times	36	67
	4-6 times	12	22
	>6 times	1	2
*Q10,* Frequency of taking sugar in coffee/tea nowadays compared to before bariatric surgery	Less frequent	26	48
	Same frequency	14	26
	More frequent	14	26
*Q11,* Eating fruits per day	None	17	31
	Once a day	30	56
	2-3 times	7	13
	>4 times	-	-
*Q12,* Frequency of eating fruits nowadays compared to before bariatric surgery	Less frequent	17	31
	Same frequency	29	54
	More frequent	8	15
*Q13,* Dietary advice from a dietician in connection with bariatric surgery	Yes	38	70
	No	16	30
*Q14,* Follow the dietician's advice immediately after bariatric surgery	Yes	30	79
	No	8	21
*Q15,* Follow the dietician's advice after bariatric surgery nowadays	Yes	12	32
	No	26	68
*Q16,* Experience of acidic reflux before bariatric surgery	Never	38	70
	Daily	5	9
	Weekly	8	15
	Monthly	3	6
*Q17,* Experience of acidic reflux after bariatric surgery	Never	11	20
	Daily	28	52
	Weekly	4	8
	Monthly	11	20
*Q19,* Experience of vomiting before bariatric surgery	Never	51	94
	Daily	-	-
	Weekly	1	2
	Monthly	2	4
*Q20,* Experience of vomiting after bariatric surgery	Never	14	26
	Daily	23	42
	Weekly	9	17
	Monthly	8	15
*Q24,* Dry mouth after bariatric surgery	Never	24	45
	Less often after surgery	7	13
	The same as before surgery	5	9
	More often after surgery	18	33
*Q25,* Feeling of discomfort after eating (dumping syndrome) after bariatric surgery	Never	7	13
	Daily	29	54
	Weekly	11	20
	Monthly	7	13
*Q26,* Overall feeling after bariatric surgery	Better than before surgery	37	69
	Worse than before surgery	12	22
	No change	3	5
	Do not know	2	4
*Q30,* Advised by a doctor to visit a dentist in connection with bariatric surgery	Yes	3	5
	No	50	93
	I don't know	1	2
*Q31,* Last dental visit	<1 year ago	41	76
	1-2 years ago	8	15
	>3 years ago	5	9
	I never visit a dentist	-	-
*Q32,* Frequency of teeth brushing	Never	9	17
	Once a day	25	46
	Twice a day	16	30
	More than twice a day	4	7
*Q33,* Use of fluoride toothpaste	Yes	34	63
	No	6	11
	I don't know	6	11
	I don't use a toothpaste	8	15
*Q34,* Use of fluoridated mouth rinse	Never	40	74
	Once a day	11	20
	Twice a day	3	6
	Once a week	-	-
	Twice a week	-	-
*Q35*, Symptoms or changes in respondents’ teeth after bariatric surgery (multiple responses per individual possible)	No symptoms	21	39
	Symptoms:	33	61
	-Teeth become yellowish	16	
	-Teeth have thinner edges	5	
	-Teeth broken off at the edges	19	
	-Increase sensation of sensitivity	15	

Questions 1-4 and 27-29 are presented in Table 1. Data from questions 6, 8, 10, 12-17, 19, 20, 24-26, 30, and 35 are discussed in the text. Questions number 18, 21-23, and 36 were omitted.

For statistical analysis, see main text.

### General description of the population

The respondents were mostly female, aged 30 to 59 years, with a university degree. The postoperative intervals ranged from 5 to more than 10 years. Their preoperative body mass index (BMI) was ≥30 (Table 1).

### Dietary habits

Numerically, most of the respondents reported less frequent intake of food and beverages after bariatric surgery (Table 2).

A significantly decreased intake of acidic drinks after bariatric surgery was reported (sign test comparing more frequent and less frequent intake, *P* < .0001).

The frequency of consumption of other products considered to increase the risk of dental erosion and caries (sugar in coffee/tea sweets, fruits) had not changed significantly (Table 2).

### Dietary advice

At the time of the bariatric surgery, 70% of respondents (n = 38) received dietary advice. Of these 38 respondents, 79% complied with the recommendations immediately after bariatric surgery, whilst fewer than half, 32%, still complied with these recommendations some years later (Table 2).

### General symptoms

More than 80% of the respondents reported postoperative symptoms related to dumping syndrome and more than 50% had daily problems (Table 2).

There was a significant increase in acidic reflux (Chi-square test comparing presence of reflux with no reflux before and after surgery, *P* = .00001) and vomiting (Chi-square test comparing presence of vomiting with no vomiting before and after surgery, *P* = .00001) after surgery. The respondents experienced these symptoms more often after bariatric surgery than before, and mostly on a daily basis (Table 2).

**Fig. 1:**
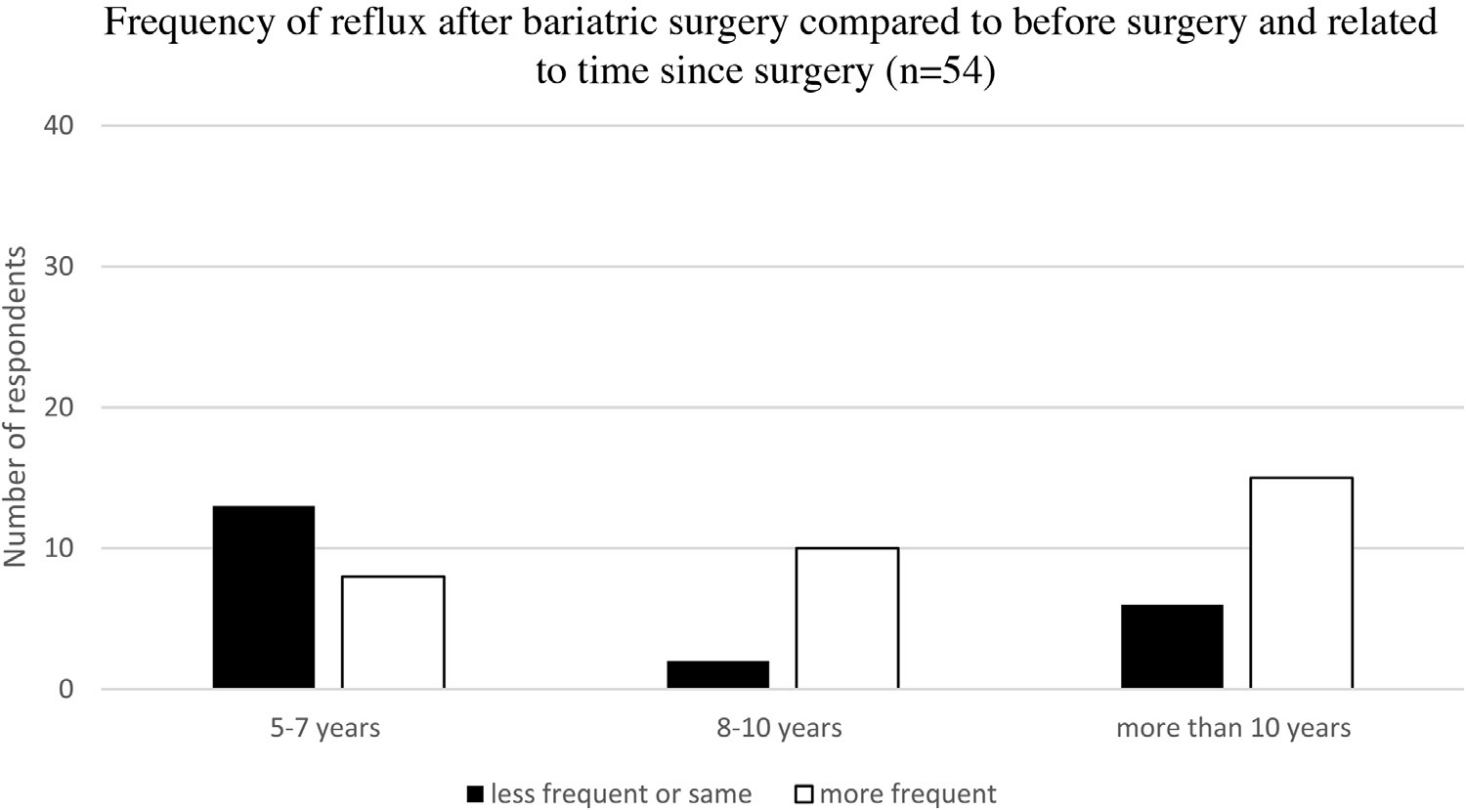
Reported frequency of reflux in relation to time after surgery. The frequency of reflux increased with time after surgery (*P* = .017292).

**Fig. 2:**
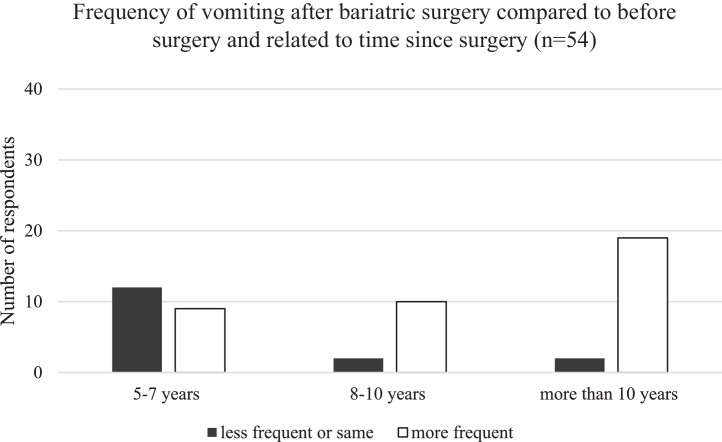
Reported frequency of vomiting in relation to time after surgery. The frequency of vomiting increased with time after surgery *(P* = .00178).

Analysis of reported frequencies of reflux and vomiting in relation to the length of time since the surgery (Figures 1, 2) showed an increase over time for both reflux (Chi-square test comparing more frequent reflux vs not more frequent reflux for 5-7, 8-10, and more than 10 years after surgery, *P* = .017292) and vomiting (Chi-square test comparing more frequent vomiting vs not more frequent vomiting for 5-7, 8-10, and more than 10 years after surgery, *P* = .00178).

**Fig. 3:**
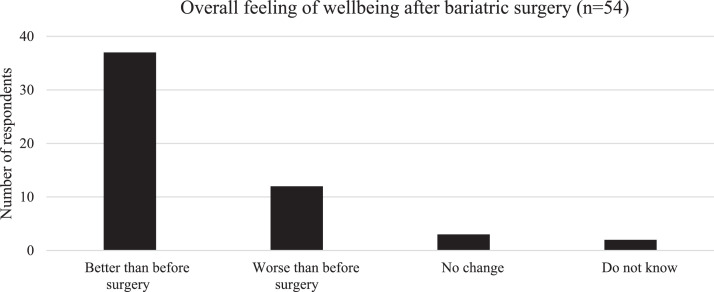
Self-reported overall feeling of well-being. Significantly more respondents reported a better overall feeling of well-being after surgery (*P* = .0006).

Most of the respondents (69%) reported that postoperatively, they experienced an improved overall feeling of well-being (sign test comparing better or worse overall feeling, *P* = .0006) (Figure 3).

### Oral symptoms

A high frequency of dental symptoms was reported after bariatric surgery. Of the 54 respondents, 33 (61%) reported 1 or more symptoms, whilst 21 (39%) reported no symptoms. The 3 main symptoms reported were yellowing of the teeth, chipping, and hypersensitivity whilst eating and drinking (Table 2). Moreover, about a third of the respondents reported a sensation of dry mouth more frequently after bariatric surgery (Table 2).

### Relationship between oral symptoms and risk factors

Respondents who reported a higher frequency of acidic reflux after bariatric surgery also reported a significantly higher frequency of oral symptoms related to dental erosion (Chi-square test correlating frequency of reflux after surgery, either more or not more, with occurrence of oral symptoms, yes or no, after surgery, *P* = .02816).

### Patient information

A vast majority of respondents reported that they were not advised to visit a dentist in connection with surgery.

## Discussion

This is, to our knowledge, the first report of long-term follow-up (more than 5 years) of patients who have undergone bariatric surgery.^18^ Most of the respondents reported general symptoms that increased over time after surgery, for example, vomiting and acidic reflux, both risk factors for dental erosion (Figures 1 and 2). It was interesting to note that in this study the respondents reported the possibility of an increased occurrence of vomiting and reflux 5 to 10 years postoperatively. In spite of this nauseating problem, respondents reported a better overall sense of well-being, indicating increased overall satisfaction with their daily life (Figure 3).^21^^,^^22^

The respondents were well educated and mostly female (Table 1), similar to previous reports^21^^,^^27^ and differing from a Saudi population mean.^28^ In this context, a higher than average educational level could hypothetically reflect several factors related to socioeconomic status: the higher socioeconomic status the better physical health.^29^ It might also reflect greater utilisation of the health care system and higher health literacy^29^^,^^30^; hence, the respondents may have been more amenable to complying with medical advice given by health professionals.

A considerable proportion (38.9%) of the respondents had undergone bariatric surgery more than 10 years ago (Table 1), suggesting that they should have had time to stabilise new habits for dietary and oral self-care routines. However, this long-term follow-up does not account for changes in surgical techniques over time. For instance, Roux-en-Y gastric bypass, which was a common method about 10 years ago, has decreased relatively in favour of gastric sleeve surgery, which was reported to be the most common method in 2016.^31^ The mechanisms underlying the weight loss achieved by the different surgical techniques may vary^32^ and the side effects could also be different.^8^ This was not taken into account in our study.

It was interesting to note that in this study as well as in other studies^16^^,^^22^ the respondents reported an increased occurrence of, for instance, vomiting, reflux, dumping syndrome, and oral symptoms, but somewhat surprisingly, despite this (Figures 1 and 2), they considered that their overall feeling of well-being had improved after surgery. This could be related to initial weight loss, physical improvement, and approaching a more ideal body image.^11^^,^^33^ The positive experiences thus seemed to override the impact of the side effects.

The respondents reported that acidic reflux and vomiting increased significantly after bariatric surgery, as has been noted previously.^34^^,^^35^ We also found a significant relationship between high frequency of acidic reflux and oral symptoms mentioned in our questionnaire. Approximately 61% of respondents experienced 1 or more oral symptoms after bariatric surgery, such as yellowing and chipping of the teeth and a sensation of hypersensitivity whilst eating and drinking. Similar studies have reported a possible association between tooth wear, gastroesophageal reflux, and bariatric surgery,^17^ as well as increased hypersensitivity of the teeth.^18^

The respondents in the present study did not report any significantly increased symptoms of oral dryness (Table 2) following bariatric surgery, which has been reported previously,^22^^,^^36^^,^^37^ as has increased salivary flow rate.^37^

Dieticians recommend that patients who have undergone bariatric surgery maintain a low-calorie intake, partly by having much smaller portions, in order to decrease the risk for dumping-related symptoms. To ensure a satisfying calorie intake, the eating frequency is increased.^38^ Previous studies have reported a drift in long-term dietary behaviour,^18^^,^^19^^,^^39^ and the risk of reverting to old habits might increase with time after surgery.

An unexpected finding was that the respondents reported a lower frequency of eating and drinking after bariatric surgery. It is possible that to some extent the respondents reverted to their previous eating pattern, as larger meals may be more satisfying. This is supported by the finding in our study, in which respondents reported complying with the dietary advice they received immediately postoperatively, but not in the long term. Similar findings are reported by Freire et al.^40^

Increased awareness about how common side effects, such as vomiting and reflux, can affect the teeth can be useful. It might increase relevant patient information from both surgeons and dieticians before and after surgery. Longer postoperative support from dieticians might help patients to avoid dietary relapse and thus the increased frequency of vomiting and reflux seen after 5 years. Increased awareness amongst dental professionals can facilitate preventive measures and early intervention if needed. It was clear from the respondents that a vast majority were not recommended to visit a dentist in connection with bariatric surgery. Medical professionals could refer bariatric surgery patients for evaluation of their oral health status before and after the surgery. In particular, the patients themselves would benefit from consultation before surgery to improve their knowledge about the risks of dental complications.

### Limitations

Lack of access to the background of the whole original sample (N = 403) made it impossible to analyse attrition. The questionnaire did not pass any transcultural translation or validation process. Together with the response rate, this might possibly limit the validity of our study.

The study design per se, using a questionnaire, might exert a limitation. It is not obvious that the recollection of habits and symptoms are correct for the respondents, or for anybody, being asked about what happened for 5, 10, or even more years ago.

Several factors have been reported to contribute to the response rate, such as the perceived relevance of the subject and the frequency of reminders.^41^ Whether the questionnaire is web-based should also be considered. However, the response rate in this study is in accordance with rates reported for web-based questionnaires.^42^^,^^43^

## Conclusions

This questionnaire study of patients’ experiences 5 to 10 years after bariatric surgery suggests a time-related, increasing occurrence of vomiting and acidic reflux: after 5 to 10 years, vomiting and reflux become even more frequent. A significant relationship was noted between a high frequency of acidic reflux and a high frequency of oral symptoms related to dental erosion. General symptoms related to dumping syndrome were common and reported by the majority of respondents to be daily occurrences. Nevertheless, in a 5- to 10-year perspective, these general symptoms and symptoms of dental erosion did not seem to detract from the respondents’ overall satisfaction with daily life.

A vast majority of respondents reported that they were not advised to visit a dentist in connection with surgery. This could possibly reduce patients’ opportunities to be given preventive measures and early interventions regarding their oral health, if needed.

## Author contributions

Dan Ericson, Kerstin Larsson and Fatimah Alsuhaibani designed the study, the questionnaire, and were responsible for data collection, data analysis and of writing the manuscript. Abdulaziz Alsuhaibani prepared the sampling and data collection and contributed to the final version of the manuscript.

## Funding

This research did not receive any specific grant from funding agencies in the public, commercial, or not-for-profit sectors.

## Conflict of interest

None disclosed.

## Appendix Supplementary materials

Image, application 1

Image, application 2

## Footnotes

Supplementary material associated with this article can be found, in the online version, at doi:10.1016/j.identj.2021.11.001.
