# Supplementary material for: Risk Factors for Dental Erosion After Bariatric Surgery: A Patient Survey
Source: Int Dent J. 2021 Dec 20;72(4):491–8. doi: 10.1016/j.identj.2021.11.001 (PMC9381372; doi:10.1016/j.identj.2021.11.001)
Supplement: Supplementary file 1 [file mmc1.docx]

# **Appendix A. Consent form (Translated from Arabic)**

You are invited to participate in a research study entitled "Impact of bariatric Surgery on Dental Erosion: A Patient Experience Study in Saudi Arabia".

Before you decide to participate, please read the following information:

**Aim of the research:**

The aim of this study is to investigate the long-term health experiences after bariatric surgery. What we are especially interested to know is if there is a connection between tooth wear and common side effect such as changes in how often and what you chose to eat and drink. We also want to investigate if you experience side effects like for instance vomiting that also can affect the teeth.

**Participants:**

A questionnaire, sent to all patients undergone bariatric surgery five years ago or more at King Saud Medical City in Riyadh, gives you an opportunity to anonymously share your experiences. The survey will be conducted through Google Form. The procedure involves filling out an online questionnaire that will take about 10 minutes. The survey consists of four parts: dietary habits, general health, dental health and oral symptoms, and its relationship to obesity surgery and dental erosion.

**Voluntary participation:**

Your decision to participate in this research is optional. If you decide to participate, you will be asked to approve this form and request to complete the online survey. If not, there will be no consequences of any kind.

**Important information:**

There are no known risks associated with this study except the amount of time spent to answer the questionnaire. The result can be useful in designing preventive programs that can help bariatric surgery patients to maintain oral health and may contribute to improve their overall quality of life.

**Confidentiality:**

Your response will be anonymous. We do not collect personally identifiable information such as your name, email address or IP address. The data collected will not be linked to the telephone number to which this request was sent. Search results will be used for scientific purposes only.

The research is approved by the Institutional Review Board (IRB) of King Saud Medical City. Please use the contact information below if you have any questions.

Dr. Fatimah Alsuhaibani

International Master student at University of Malmö, Sweden

[Aj5200@student.mau.se](mailto:Aj5200@student.mau.se)

ELECTRONIC CONSENT: Please select your choice below.
Clicking on the "agree" button below indicates that: 
• you have read the above information.
• you voluntarily agree to participate.
If you do not wish to participate in the research study, please decline participation by clicking on the "disagree" button.

Agree

Disagree
