# Supplementary material for: Risk Factors for Dental Erosion After Bariatric Surgery: A Patient Survey
Source: Int Dent J. 2021 Dec 20;72(4):491–8. doi: 10.1016/j.identj.2021.11.001 (PMC9381372; doi:10.1016/j.identj.2021.11.001)
Supplement: Supplementary file 2 [file mmc2.docx]

# **Appendix B. Questionnaire (Translated from Arabic)**

**Demographic characteristics**

1. Gender:

- Male
- Female

2. Age:

- Under 20 years
- 20-29 years
- 30-39 years
- 40-49 years
- 50-59 years
- 60 years or more

3. What is the highest educational degree you have achieved?

- Primary school degree
- Middle school degree
- High school degree
- Diploma degree
- University degree (bachelor’s degree)
- Higher education degree (master, doctoral etc)

4. How long ago did you undergo bariatric surgery?

- 5-7 years
- 8-10 years
- More than 10 years

**Dietary habits**

5.How many times per day do you eat or drinks? (drinking water not included)

- 1-2 times
- 3-4 times
- 5-6 times
- More than 6 times

6. How often do you eat, or drinks nowadays compared to before bariatric surgery?

- More frequent
- Less frequent
- Same frequency

7. How many times per day do you drink acidic drinks for example water or tea with lemon/soft drinks/energy drinks/juices?

- None at all
- 1-3 times
- 4-6 times
- More than 6 times

8. How often do you drink acidic drinks for example water or tea with lemon/soft drinks/energy drinks/juices nowadays, compared to before bariatric surgery?

- More frequent
- Less frequent
- Same frequency

9. How many times per day do you take sugar in coffee/tea or eat sweets?

- None
- 1-3 times
- 4-6 times
- More than 6 times

10. How often do you take sugar in coffee/tea or eat sweets nowadays compared to before bariatric surgery?

- More frequent
- Less frequent
- Same frequency

11. How many times per day do you eat fruit?

- None
- Once a day
- 2-3 times
- More than 4 times

12. How often do you eat fruits nowadays compared to before bariatric surgery?

- More frequent
- Less frequent
- Same frequency

13. Did you get any dietary advice from a dietician in connection with your bariatric surgery? (If “Yes”, continue with question number 14 and question number 15, if “No”, go directly to question number 16)

- Yes
- No

14. Did you follow the dietician’s advice immediately after bariatric surgery?

- Yes
- No

15. Do you follow the dietician’s advice after bariatric surgery nowadays?

- Yes
- No

**General health**

16. Did you have experience from acidic reflux before bariatric surgery?

- Daily
- Weekly
- Monthly
- Never

17. Do you have experience from acidic reflux after bariatric surgery?

- Daily
- Weekly
- Monthly
- Never

18. How often do you experience acidic reflux after bariatric surgery?

- More often after surgery
- Less often after surgery
- The same as before surgery
- Never

19. Did you have experience from vomiting before bariatric surgery?

- Daily
- Weekly
- Monthly
- Never

20. Do you have experience from vomiting after bariatric surgery?

- Daily
- Weekly
- Monthly
- Never

21. How often do you vomit after bariatric surgery?

- More often after surgery
- Less often after surgery
- The same as before surgery
- Never

22. Did you have experience from dry mouth before bariatric surgery?

- Daily
- Weekly
- Monthly
- Never

23. Do you have experience from dry mouth nowadays?

- Never
- Seldom
- Often

24. How often do you experience dry mouth after bariatric surgery?

- More often after surgery
- Less often after surgery
- The same as before surgery
- Never

25. After bariatric surgery do you feel uncomfortable (for example: nausea, vomiting, too full, abdominal cramps, diarrhea, dizziness, rapid heart rate) after eating?

- Daily
- Weekly
- Monthly
- Never

26. How is your overall feeling after bariatric surgery?

- Better than before surgery
- Worse than before surgery
- No change
- Do not know

**Length and weight before and after**

27. What is your length….. cm

28. What was your weight before bariatric surgery …. kg

29. What is your weight now….kg

30. In connection with bariatric surgery, did your doctor advice you to visit a dentist?

- Yes
- No
- I don’t know

**Dental habits**

31. When was your last dental visit?

- Less than 1 year ago
- 1-2 years ago
- More than 3 years ago
- I never visit a dentist

32. How often do you brush your teeth?

- Never
- Once a day
- Twice a day
- Once a week
- Twice a week

33. Do you use fluoride toothpaste?

- Yes
- No
- I don’t know

34. How often do you use fluoridated mouth rinse?

- Never
- Once a day
- Twice a day
- Once a week
- Twice a week

**Oral symptoms**

35. Did you or your dentist notice any changes in your teeth after bariatric surgery? (you can choose more than one alternative)

- No
- My teeth have become yellowish
- My teeth have become smoother, brighter surface
- My teeth have thinner edges
- My teeth have small pieces broken off at the edges
- My teeth look shorter
- An increased sensation of pain or sensitivity when I drink hot/cold liquids or eating acidic/sweet foods
- Other, please describe…….

36. Do you want to describe any other significant symptom or feeling that you experienced after bariatric surgery?
